# An update on clinical trials for cutaneous lupus erythematosus

**DOI:** 10.1111/1346-8138.17161

**Published:** 2024-03-15

**Authors:** Lillian Xie, Lais Lopes Almeida Gomes, Caroline J. Stone, Daniella Forman Faden, Victoria P. Werth

**Affiliations:** ^1^ Corporal Michael J. Crescenz Veterans Affairs Medical Center Philadelphia Philadelphia Pennsylvania USA; ^2^ Department of Dermatology School of Medicine, University of Pennsylvania Philadelphia Pennsylvania USA

**Keywords:** anifrolumab, clinical trials, cutaneous lupus, iberdomide, litifilimab

## Abstract

Cutaneous lupus erythematosus (CLE) comprises dermatologic manifestations that may occur independently or with systemic lupus erythematosus (SLE). Despite advancements in refining CLE classification, establishing precise subtype criteria remains challenging due to overlapping presentations and difficulty in distinguishing morphology. Current treatments encompass preventive measures, topical therapies, and systemic approaches. Hydroxychloroquine and glucocorticoids are the sole US Food and Drug Administration (FDA)‐approved medications for CLE, with numerous off‐label treatments available. However, these treatments are often not covered by insurance, imposing a significant financial burden on patients. The exclusion of most CLE patients, particularly those without concurrent SLE, from trials designed for SLE has resulted in a lack of targeted treatments for CLE. To develop effective CLE treatments, validated outcome measures for tracking patient responsiveness are essential. The Cutaneous Lupus Erythematosus Disease Area and Severity Index is widely utilized for its reliability, validity, and ability to differentiate between skin activity and damage. In contrast, the FDA mandates the use of the Investigator's Global Assessment, a five‐point Likert scale related to lesion characteristics, for skin‐related therapeutic trials. It requires the disease to resolve or almost completely resolve to demonstrate improvement, which can be difficult when there is residual erythema or incomplete clearance that is meaningfully improved from a patient perspective. Various classes of skin lupus medications target diverse pathways, allowing tailored treatment based on the patient's lupus inflammatory profile, resulting in improved outcomes. Promising targeted therapeutic drugs include anifrolumab (anti‐type 1 interferon), deucravacitinib (allosteric tyrosine kinase 2 inhibitor), litifilimab (plasmacytoid dendritic cell‐directed therapy), iberdomide (cereblon‐targeting ligand), and belimumab (B‐cell directed therapy). Despite the significant impact of CLE on quality of life, therapeutic options remain inadequate. While promising treatments for cutaneous lupus are emerging, it is crucial to underscore the urgency for skin‐focused treatment outcomes and the implementation of validated measures to assess therapeutic effectiveness in clinical trials.

## 1 CLINICAL LANDSCAPE

Cutaneous lupus erythematosus (CLE) is an autoimmune skin disease with dermatologic manifestations that can occur with or without systemic lupus erythematosus (SLE). CLE lesions typically involve sun‐exposed areas secondary to photosensitivity. Disease chronicity coupled with the potential for long‐lasting secondary sequelae can significantly impact quality of life for affected individuals. CLE patients are screened at diagnosis with clinical and laboratory tests and continue to be monitored at least annually.

The Gilliam and Sontheimer classification system for CLE was established in 1982 and describes three major subtypes as umbrella categories for more specific categorizations: acute CLE (ACLE), subacute CLE (SCLE), and chronic CLE (CCLE) which includes discoid LE (DLE).^1^ While subtypes are often distinguishable by clinical features, absolute subtype criteria can be challenging due to overlap in presentation and difficulty in distinguishing morphology.^2^ Distinguishing features, such as atrophy and scarring in DLE, may present later in the disease course and can be difficult to discern clinically.

Despite the clinical variation among different subtypes, the strategies for management often overlap. Existing treatments target disease through prevention, topical therapies, and systemic measures. Preventative strategies primarily focus on avoidance of common triggers through SPF sun protection greater than 70, smoking cessation, and minimizing exposure to photosensitizing medications.^3^ For cases with limited dermatologic involvement, topical therapies such as topical corticosteroids or calcineurin inhibitors directly target active lesions with minimal risk for systemic effects.

Remarkably, there have been no new US Food and Drug Administration (FDA)‐approved systemic treatments for CLE in over 60 years. The only FDA‐approved medications for CLE are hydroxychloroquine and glucocorticoids, which were accepted under previous regulations before implementation of the current standards for clinical trials. Off‐label therapies include other antimalarials such as chloroquine and quinacrine as well as immunosuppressants, such as methotrexate and mycophenolate mofetil. Additional treatment options include dapsone, retinoids (acitretin, isotretinoin, and alitretinoin), thalidomide, and lenalidomide. Treatments approved for SLE are sometimes used for CLE treatment.^4^ Because they are considered off‐label treatments, these medications are not usually covered by insurance companies, placing a significant burden on the patient. This category includes medications such as rituximab, belimumab, and anifrolumab.

## 2 PATHOGENESIS

Understanding the current landscape for CLE drug development requires a brief overview of CLE pathogenesis. Some variation exists between different subtypes, for example there is on average a deeper and denser infiltrate in DLE compared to SCLE. Despite these differences, chronic inflammation driving a positive feedback loop involving both the innate and adaptive immune systems is common to all subtypes. The cycle begins with an environmental trigger such as ultraviolet (UV) light exposure, smoking, or certain pharmacologic agents, leading to keratinocyte apoptosis and chemokine production throughout the epidermis.^5^ This process promotes recruitment of proinflammatory cells. Release of intracellular debris signals recruitment of autoreactive T cells, plasmacytoid dendritic cells (pDCs), and B cells through interactions between danger‐associated molecular patterns and their associated pattern recognition receptors.^6^ Ultimately, more research is needed to understand the specific genetic, epigenetic, and environmental variables that produce different clinical phenotypes.

Interferon (IFN)‐1 elevation is characteristic of SLE, although detection through enzyme‐linked immunosorbent assay has been historically challenging.^7^ Instead, IFN‐1 levels have been measured by proxy through quantification of IFN‐induced genes and have been positively correlated with clinical activity scores^8^, ^9^ This hypothesis, in conjunction with the existing knowledge that pDCs can produce copious amounts of IFN‐1 in response to viral stimuli, has led to a widespread assumption that pDCs are the major producers of the elevated IFN‐1 in SLE.^10^, ^11^

However, emerging research has suggested otherwise. Even though IFN‐α and IFN‐responsive genes are upregulated both in the blood and in the skin, Psarras et al. have shown that SLE pDCs are incapable of producing IFN‐1 in response to toll‐like receptor (TLR)‐7 or TLR‐9 stimulation.^12^ Through immunoprofiling of SCLE and DLE, Vazquez et al. further challenged the existing dogma and determined that pDCs are not major producers of type I IFN when measured through heatmaps of single cell pathway expression in pDCs derived from imaging mass cytometry, confirmed by messenger ribonucleic acid (mRNA) in situ hybridization and suspension.^7^ Even with stimulation, CLE pDCs do not produce substantial IFN‐1 ex vivo, as determined through quantification via flow cytometry.^7^ These data support that CLE pDCs in lesional skin and peripheral blood do not produce IFN‐1 in their native environment and are relatively incapable of producing IFN‐1 on TLR‐7 stimulation ex vivo. The role of pDCs and IFNs in the pathogenesis of CLE is an area in need of further investigation.

While the exact role of pDCs and IFNs remains elusive, recent CLE investigational treatments targeting intracellular signaling and inflammatory pathways including the IFN‐1 cascade demonstrate promising results. Investigational targets include inhibitors directed toward different molecules implicated in CLE pathogenesis, which will be discussed in later sections.

## 3 OUTCOME MEASURES

A critical review of the clinical trial methodologies used in CLE lupus therapeutic development reveals significant gaps, particularly in the outcome measures used to evaluate SLE and their applicability to CLE. The primary outcome measures, including the Systemic Lupus Erythematosus Disease Activity Index (SLEDAI)^13^ and the British Isles Lupus Assessment Group Index (BILAG),^14^ are primarily designed for systemic manifestations. BILAG captures partial change over time with some skin measures like skin eruption and alopecia, while SLEDAI documents presence or absence of organ involvement, including skin features such as new rash and mucosal ulcers. In response to these limitations, composite outcome measures such as the SLE Responder Index (SRI) and BILAG‐Based Combined Lupus Assessment (BICLA) were developed.^15^ However, these too rely heavily on systemic disease measures, perpetuating a clear inadequacy in addressing the specific needs of CLE patients.

The minimization of skin concerns is further compounded by SLE trial entry criteria. Historically, SLE clinical trials have framed inclusion guidelines based on criteria from the American College of Rheumatology (ACR) or the Systemic Lupus International Collaborating Clinics (SLICC), which do not require positive serology.^16^, ^17^ However, the 2019European Alliance of Associations for Rheumatology (EULAR)/ACR classification criteria necessitate a positive antinuclear antibody (ANA) for diagnosis, excluding a significant portion of CLE patients, particularly the 18% with a negative ANA, who would have qualified under previous criteria.^18^ This reliance on serology, coupled with the poor standardization of ANA testing, makes entry into clinical trials problematic for many CLE patients.^19^

This systematic exclusion is not just a matter of classification but has real‐world implications. The majority of CLE patients, who may not have concomitant SLE, are excluded from the trials designed for SLE, leading to a lack of targeted treatments for CLE.^20^ Despite the considerable impact of CLE on patients' quality of life, the therapeutic options remain inadequate. It is imperative that we establish targeted investigations to address and alleviate the unique challenges faced by CLE patients.

To develop effective CLE treatments, validated outcome measures to track patient responsiveness are essential. The Cutaneous Lupus Erythematosus Disease Area and Severity Index (CLASI) stands out as a comprehensive tool, widely used for its reliability, validity, and ability to differentiate between skin activity and damage.^21^, ^22^, ^23^ It captures the granularity of CLE activity across anatomical distribution and correlates with improvements in quality of life. Despite its extensive application in SLE trials and in lupus literature, the CLASI has yet to be endorsed by the FDA as a primary endpoint.

Instead, the FDA mandates use of a 5‐point Likert scale, the Investigator's Global Assessment (IGA), for skin‐related therapeutic trials.^24^ However, its application to CLE is met with challenges due to the need to define a successful outcome as clear or almost clear skin, which is often an unrealistic goal for CLE patients, when residual erythema may not resolve quickly and patients are meaningfully improved with lesser degrees of improvement.^25^, ^26^ Emerging evidence suggests that a 50% reduction in the CLASI score from baseline, known as CLASI‐50, correlates with significant quality of life improvements.^26^ The CLE‐IGA's stringent criteria may not capture these changes, which can result in a failed clinical trial even if a medication has a meaningful impact from the patient perspective.^27^ At the current time, the dermatology community agree that the CLASI is best as a primary outcome, with other outcomes such as CLE‐IGA and quality of life measures being important secondary outcomes that require further validation.^25^, ^26^

## 4 ONGOING TRIALS

### 4.1 IFN‐targeting therapies

#### 4.1.1 Anifrolumab

Among IFN‐directed therapies, anifrolumab is the most studied. Anifrolumab is a humanized immunoglobulin (IgG)1κ monoclonal antibody that acts via blockade of the IFN‐α/β/ω receptor (IFNAR) to inhibit signaling of all five type I IFNs, that is, IFNα, IFNβ, IFNɛ, IFNκ, and IFNω.^28^, ^29^ Disruption of the IFNAR complex assembly then suppresses the immune dysfunction and tissue injury associated with flaring disease.

Previous data generated in one phase II (MUSE) and two phase III (TULIP‐1, TULIP‐2) clinical trials prompted FDA approval of anifrolumab for SLE patients in 2021.^30^ In the phase II trial, patients with baseline CLASI activity (CLASI‐A) score ≥10 who received anifrolumab had a higher relative reduction of Systemic Lupus Erythematosus Activity Index 2000 (SLEDAI‐2K) scores and decreased overall corticosteroid dosage at the primary endpoint of 24 weeks compared to placebo.^29^ At the secondary endpoint of 52 weeks, 63% of patients in the 300‐mg arm and 58.3% of patients in the 1000‐mg arm demonstrated a ≥50% improvement in CLASI‐A as compared to placebo (30.8%).^29^ TULIP‐1 did not meet its primary endpoint using the Systemic Lupus Erythematosus Responder Index (SRI)‐4, whereas TULIP‐2 met its endpoint using the BICLA.^31^ A greater percentage of TULIP‐2 patients treated with anifrolumab had a ≥50% improvement in CLASI‐A (49%) compared to placebo (25%, adjusted difference = 24%, confidence interval [CI] 4.3–43.6, *P* = 0.04). A 3‐year extension of TULIP‐2 demonstrated a favorable long‐term safety profile, significant reduction in overall usage of steroids, and improved SLEDAI‐2K scores compared to placebo.^32^ However, skin was not followed as a marker of clinical improvement.

Since the approval of anifrolumab in SLE, various case studies and case series have supported its effectiveness for refractory CLE and mucocutaneous manifestations in SLE.^33^, ^34^, ^35^, ^36^ However, additional studies are necessary to optimize dosing, characterize response rates, and ascertain long‐term effects. Although these studies demonstrate progress in treating CLE in SLE patients, it remains necessary to show trial data that establishes efficacy in patients with CLE alone. Furthermore, patients suffering from CLE in the absence of SLE may encounter barriers in accessing these novel therapies due to strict insurance policies that require evidence of systemic involvement for coverage approval. Ultimately, while the success of anifrolumab paved the way for development of new treatments targeting the IFN‐I pathway, further investigation is needed to include CLE patients. Planning for such a study is now underway.

### 4.2 Janus kinase/signal transducers and activators of transcription (JAK/STAT) inhibitors

#### 4.2.1 Deucravacitinib

Deucravacitinib is a selective oral, allosteric tyrosine kinase (TYK)2 inhibitor that suppresses downstream effects modulated by IFN‐I, interleukin (IL)‐10, IL‐12 and IL‐23.^37^ Following two phase III trials, deucravacitinib was approved for the treatment of plaque psoriasis in 2022 without the US black box warning found in other JAK inhibitors.

In PAISLEY, a phase II trial in SLE for oral deucravacitinib, patients were randomized in a 1:1:1 ratio to receive placebo or one of three possible dosing regimens, 3 mg twice a day (BID), 6 mg BID, or 12 mg once a day (qD), for 48 weeks.^37^ The primary endpoint measuring SRI‐4 response rates was met at week 32. At the secondary endpoint of week 48, subjects with a baseline CLASI‐A ≥ 10 demonstrated a CLASI‐50 response of 16.7% in placebo versus 69.6% in the 3 mg BID deucravacitinib group (odds ratio [OR] 10.5, *P* < 0.001). Secondary efficacy endpoints, including SRI‐4, BICLA, and Lupus Low Disease Activity State (LLDAS), were assessed at week 48. All outcome measure thresholds demonstrated superior response rates in patients in the 3 mg BID treatment arm compared to placebo. While secondary efficacy endpoint results were higher in the 6 mg BID and 12 mg qD groups compared to placebo, the results were not statistically significant. Skin‐specific endpoints favored deucravacitinib 3 mg BID over placebo (CLASI‐50 response, 69.6% vs 16.7%, respectively, *P* < 0.001 vs placebo). The most frequently reported adverse events included upper respiratory and urinary tract infections, nasopharyngitis, and headaches.^37^ Notably, all doses of deucravacitinib resulted in decreased IFN gene expression through week 44 compared to placebo. Currently, there are two phase III studies for deucravacitinib in SLE underway (NCT05617677 and NCT05620407). A phase II trial in CLE, specifically in patients with active DLE/SCLE, is also ongoing (NCT048570340).

#### 4.2.2 Baricitinib

Baricitinib is a JAK1/2 inhibitor currently approved for alopecia areata. Two phase III trials (SLE‐BRAVE‐I and SLE‐BRAVE‐II) demonstrated mixed efficacy for treatment of SLE. SLE‐BRAVE‐I met its primary endpoint, showing reduction of SRI‐4 and improvement of mucocutaneous symptoms in the 4 mg group (57%, OR 1.57, 95% CI 1.09–2.27, difference with placebo 10.8 (2.0–19.6), *P* = 0.016) compared to placebo (46%).^38^ No secondary outcomes were met. In the identically designed SLE‐BRAVE‐II, the proportion of SRI‐4 responders at week 52 in the 4 mg treatment arm (47%; OR 1.07, 95% CI 0.75–1.53; difference with placebo 1.5, 95% CI −7.1 to 10.2; *P* = 0.71) and 2 mg treatment arm (46%, OR 1.05, 95% CI 0.73–1.50, difference with placebo 0.8, 95% CI −7.9 to 9.4; *P* = 0.79) was not significantly different compared to placebo (46%).^39^ No major secondary endpoints, including time to first severe flare and glucocorticoid tapering, were met.

#### 4.2.3 Tofacitinib

An open‐label, single‐center phase II pilot study investigating tofacitinib for DLE was discontinued due to low recruitment. Of the five DLE patients who received 5 mg of oral tofacitinib BID for 24 weeks, two showed CLASI‐A score improvements of 75% by week 24 and two were less responsive, with a 30% improvement at weeks 16 and 24.^40^ The results of the phase Ib/II trial in CLE are not yet published (NCT03288324).

#### 4.2.4 Filgotinib and lanraplenib

Like JAKs, spleen tyrosine kinases are implicated in inflammatory cytokine synthesis in CLE patients. In a phase II, randomized, double‐blind, trial investigating filgotinib and lanraplenib each compared to placebo, the primary endpoint of a 12‐week difference in CLASI‐A from baseline was not met for either investigational treatment.^41^

#### 4.2.5 Delgocitinib

A phase II trial (NCT03958955) examining delgocitinib, a TYK2 and JAK1/2/3 inhibitor, was aborted due to low recruitment. The primary endpoint was not met at week 6, measured as the percentage of target lesions with an IGA score of 0 (clear) or 1 (almost clear) compared to placebo.^42^ However, a case report showed successful treatment of SCLE on the face with delgocitinib 0.5% ointment, a commonly used topical for atopic dermatitis.^43^

#### 4.2.6 Ruxolitinib

An open‐label phase II trial evaluating topical ruxolitinib 1.5% cream in DLE is ongoing (NCT04908280). Subsequent case reports showed improvement in CLE lesion severity, and in vitro CLE models showed decreased cytokine production of CXCL10, CXCL9, and MxA.^44^, ^45^

### 4.3 pDC‐directed therapies

#### 4.3.1 Litifilimab

Litifilimab is a promising pDC‐directed therapy.^46^ This humanized IgG1 antibody binds to blood dendritic cell antigen 2 (BDCA2), a receptor specific to pDC surfaces.^46^, ^47^ It inhibits pDC activation through fragment crystallizable (Fc)‐region‐dependent and independent mechanisms, which then results in decreased production of all type I and III IFNs, IL‐6, and a number of chemokines.^48^, ^49^, ^50^ Phase I included 54 healthy controls and 12 SLE patients.^51^ Single doses of litifilimab showed favorable safety and pharmacokinetics, with serum drug concentrations correlating with internalization of BDCA2 on pDCs and dampened expression of IFN. Subjects treated with litifilimab also showed reductions in CLASI‐A and skin IFN‐induced MxA expression. Six of the eight SLE patients who received a single dose of litifilimab had a reduction in CLASI‐A score of at least 4 points at week 4 and/or week 12, which was also supported by reductions in MxA levels on lesional biopsy.

Phase II (LILAC) was a two‐part, multicenter placebo‐controlled trial that examined the efficacy of litifilimab in SLE for Part A (NCT02847598) and in CLE for Part B (NCT02847598).^52^ LILAC is groundbreaking as an investigational trial, not only in its treatment efficacy for SLE and CLE, but also in its separate investigation of systemic and cutaneous disease, which allows investigators to focus on meaningful changes in skin disease activity.

In Part A, 110 SLE patients were randomized to receive either litifilimab 450 mg or placebo. Primary analysis included examining the 24‐week change in count of swollen/tender joints relative to baseline. The litifilimab group demonstrated a greater decrease from baseline in the number of active joints (19.0 ± 8.4) in contrast to the placebo group (21.6 ± 8.5). Secondary endpoints included the CLASI‐50 response at week 24, the change in CLASI‐A score from baseline measured at weeks 12, 16, and 24, and the percentages of patients with CLASI‐A score improvement of at least 4 and at least 7 at week 24. Among these endpoints, all but one did not reach statistical significance in evaluating between‐group differences.

Part B recruited 132 SCLE and/or DLE patients with moderate‐to‐severe disease activity with or without systemic manifestations.^52^ At the start, Part B enrolled 31 participants for a single‐dose efficacy design in a 2:1 ratio of 450 mg litifilimab to placebo. Because of slow recruitment and to evaluate litifilimab across doses, the protocol was modified to a dose‐ranging trial with two additional litifilimab doses, 50 and 150 mg, and enrollment expanded to 130 patients over 16 weeks. Patients were randomly and equally distributed to receive placebo or one of three dosing regimens: 50, 150, or 250 mg. The primary endpoint was measured at 16 weeks as percentage change in CLASI‐A from baseline with a dose–response model. There was a significant difference in CLASI‐A improvement in all treatment arms compared to placebo, with notable decreases of −24.3% (95% CI −43.7 to −4.9) in the 50 mg arm, −33.4% (95% CI −52.7 to −14.1) in the 150 mg arm, and −28.0% (95% CI −44.6 to −11.4) in the 450 mg arm. Subsequent analysis using the least‐squares mean changes to create a dose–response model revealed a statistically significant effect. Since this study did not have the statistical power to effectively evaluate secondary endpoints, most of the 95% CIs comparing least‐squares mean differences between litifilimab doses and placebo included zero and therefore did not corroborate the primary analysis findings.

A two‐part phase II Part A/phase III Part B multicenter, randomized, double‐blind study (AMETHYST, NCT05531565) studying litifilimab in SCLE or Chronic Cutaneous Skin Lupus (CCLE) is currently underway and has begun recruitment. Primary outcome measures include Cutaneous Lupus Activity of Physician's Global Assessment‐Revised (CLA‐IGA‐R) score and CLASI‐A score.

#### 4.3.2 Daxdilimab

Another pDC‐specific approach with promising results is daxdilimab, a monoclonal antibody that depletes pDCs by binding to immunoglobulin‐like transcript 7 (ILT7).^53^ A phase I study of daxdilimab showed CLASI‐A improvement that correlated with substantially reduced skin pDCs and decreased IFN‐I activity without increased incidence of infection. A phase II trial in moderate‐to‐severe DLE is ongoing (NCT05591222).

### 4.4 Cereblon‐targeting ligands

#### 4.4.1 Iberdomide

Iberdomide is a high‐affinity ligand of cereblon that inhibits the anti‐cullin‐Really Interesting New Gene (RING)‐E3 ubiquitin ligase 4 complex.^54^ By binding to cereblon, iberdomide induces polyubiquitination followed by proteasomal degradation of Ikaros (IKZF1) and Aiolos (IKZF3). Decreased Ikaros and Aiolos protein levels lead to the suppression of pDCs and the IFN‐I response, the reduction of B cells and anti‐dsDNA antibodies, and the increased production of IL‐2 and Treg cells.^55^

A randomized, placebo‐controlled phase II trial examined safety and efficacy of iberdomide in patients with active SLE.^56^ Patients were randomly and equally distributed to receive doses of iberdomide or placebo. The primary endpoint at week 24 was met, with SRI‐4 achieved in 54% of those who received iberdomide 0.45 mg compared to 35% of those who received placebo. SRI‐4 responses were greater in patients with a high type 1 IFN or Aiolos gene signature. The proportion of SRI‐4 responders was maintained or improved among all dosing groups over 52 weeks. At week 24, an increased proportion of patients in the placebo group achieved SRI‐4 after switching to iberdomide. Secondary outcomes examined CLASI‐50 in patients with a baseline CLASI‐A ≥ 10. When compared to placebo, the differences in CLASI‐50 responders for those receiving iberdomide 0.45, 0.30, and 0.15 mg were 14.2%, 5.3%, and 24.0% respectively. The distribution of CLE subtypes was 56% ACLE, 29% CCLE, and 16% SCLE. Notably, the 0.45‐mg dose of iberdomide showed a differential CLASI‐50 response, with a 38.7% difference from placebo in SCLE cases (*P* = 0.035) and a 34.1% difference in CCLE cases (*P* = 0.029), in contrast to a −3.0% difference in ACLE cases (*P* = 0.782). At present, there are no plans for a phase III trial.

### 4.5 B‐cell therapies

#### 4.5.1 Belimumab

Belimumab is a monoclonal B cell‐activating factor (BAFF) receptor antagonist that is the most promising among the B‐cell directed therapies. It is a B‐cell inhibitor used for diffuse and treatment‐resistant CLE in the setting of SLE.^57^ In 2022, Manzi et al. conducted a pooled post hoc analysis of five phase III randomized control trials to investigate the effects of belimumab on skin, specifically BLISS‐76, BLISS‐52, North East Asia, BLISS‐SC, and EMBRACE.^58^, ^59^, ^60^, ^61^, ^62^ Among the 3086 patients included for the analysis (belimumab, *n* = 1869; placebo, *n* = 1217), a majority of patients had mucocutaneous findings at baseline (SELENA‐SLEDAI 85%, BILAG category A or B 60%), with 8% (SELENA‐SLEDAI) and 9% (BILAG) who had vasculitis manifestations.^63^ At week 52, belimumab patients experienced improvements in mucocutaneous domains for SELENA‐SLEDAI (59.4% vs 49.2%, treatment difference from placebo 10.3%, *P* < 0.0001) and BILAG (54.1% vs 43.1%, treatment difference from placebo 11.0%, *P* < 0.0001). Additionally, belimumab showed statistically significant improvement over placebo for four SELENA‐SLEDAI metrics (vasculitis, rash, alopecia, and mucosal ulcers) and nine BILAG categories. Of the SELENA‐SLEDAI items, the largest treatment difference in favor of belimumab over placebo was for vasculitis (25.4%, *P* < 0.0001). Of the BILAG items, the greatest treatment difference was for cutaneous vasculitis (34.1%, *P* = 0.0039).

A recent systematic review and meta‐analysis demonstrated a 44%–55% reduction in flare rates for CLE patients with or without SLE over the course of 1 year while on belimumab treatment. Overall, belimumab requires 20 weeks to elicit a significant clinical response, with the peak effect observed at 1 year. A phase III, multicenter 24‐week trial (BELI‐SKIN, EUDRA‐CT: 2017‐003051‐35) is now underway to investigate the efficacy of belimumab for refractory skin manifestations with the primary endpoint of relative change in mean Revised CLASI (RCLASI) activity score at 24 weeks between placebo and treatment arms.

#### 4.5.2 Rituximab

Rituximab is a monoclonal antibody that targets B cells expressing CD20. Previous trials investigating its efficacy for moderate‐to‐severe SLE have shown mixed efficacy as a therapy for skin lesions. A multicenter, randomized, placebo‐controlled phase II trial investigated rituximab versus placebo by BILAG responsiveness. Baseline disease characteristics were categorized into different SLE domains, but were not separately analyzed in the primary or secondary analysis. The rituximab group did not demonstrate any significant difference from the placebo group at either endpoint. Adverse events, namely serum sickness and neutropenia, were more frequent in the rituximab group, although patients in both cohorts continued background immunosuppressives and prednisone during the trial.^64^

Several subsequent studies continued to study its efficacy in SLE patients with skin activity. In a prospective study of 82 patients with SLE, mucocutaneous response was measured in the 26 patients with baseline skin activity captured by the BILAG. Clinical responsiveness measured at 6 months showed improvement in a greater proportion of ACLE patients (6 of 14) than CCLE patients (0 of 8, *P* = 0.034).^65^ Interestingly, 12 SLE patients who either had no baseline cutaneous activity or who presented with ACLE experienced new‐onset CCLE or SCLE flares following treatment with rituximab.^65^ A 2018 single‐center, retrospective, cohort study tested clinical responsiveness at 6 and 12 months with BILAG grading. At 6 months, 38 of 50 (76%) participants showed improvement from their status of BILAG A or B scores. Of the 46 patients available for follow up at 12 months, 28 (61%) showed a clinical response, with 12 of 21 ACLE (57%) and 6 of 10 nonspecific lupus erythematosus (60%) showing the highest response rates.^66^ While rituximab is an important tool in the SLE armamentarium, its role in CLE appears less defined and requires further investigation.

### 4.6 T‐cell therapies

#### 4.6.1 Dapirolizumab

Dapirolizumab is an antibody fragment that targets the CD40 ligand (CD40L) typically found on activated T cells and platelets.^67^ Compared to a prior anti‐CD40L prototype, dapirolizumab carries a lower risk of thromboembolism due to the absence of the Fc domain normally involved in platelet aggregation.^68^ In a randomized, placebo‐controlled, phase II study of dapirolizumab for SLE, the 24‐week BICLA responder rates did not fit the predetermined dose–response relationship (*P* = 0.07) and thus fell short of the primary endpoint of identifying a dose–response relationship. Secondary endpoints included other outcome measures such as CLASI scores. Notably, patients treated with dapirolizumab experienced greater improvements from baseline CLASI activity scores (dapirolizumab 6 mg/kg −4.5 [standard deviation, SD = 6.3], dapirolizumab 24 mg/kg −4.7 [5.5], dapirolizumab 45 mg/kg −5.8 [5.2]) compared to placebo (placebo −2.9 [5.4]) at week 24. At week 48, the dapirolizumab dosing groups continued to show superiority in CLASI‐A improvement over placebo (dapirolizumab 6 mg/kg −5.0 [6.1], 24 mg/kg −4.6 [5.5], 45 mg/kg −6.8 [6.0], placebo −3.6 [4.1]). While rates of infection were higher in dapirolizumab‐treated patients than placebo, a heightened risk of thromboembolism was not observed. A phase III trial in SLE is active and awaiting recruitment (NCT04294667), without skin‐specific primary or secondary outcome measures.

#### 4.6.2 Lulizumab

Lulizumab is a monoclonal antibody that targets CD28, a critical costimulatory T‐cell molecule.^69^ By blocking CD28, lulizumab prevents activation of aberrant T cells mediating autoimmune disease. A multicenter, double‐blind phase II trial of lulizumab did not meet its primary endpoint of BICLA response rates at week 24 or its secondary endpoints, which included CLASI‐20 and CLASI‐50. Among 349 SLE patients randomized across five treatment arms, no significant differences in skin‐specific or systemic outcome measures were observed between these groups.

### 4.7 Other targets

#### 4.7.1 Etanercept

Etanercept is a TNF‐α (tumor necrosis factor alfa) inhibitor that is administered intradermally, which may be sufficient to treat active lesions with a lowered risk for systemic TNF effects.^70^ Such effects have been thoroughly established in the literature and include induction of pathogenic autoantibodies, initiation of a disease flare, and risk of drug‐induced SCLE.^71^, ^72^, ^73^ A single‐arm phase II trial (NCT02656082) recruited 25 DLE patients, with a primary endpoint at week 12 of at least six participants reaching a 20% decrease from baseline in the modified limited Score of Activity and Damage in DLE (ML‐SADDLE). Of the total recruited participants, 17 completed the primary efficacy assessment, with 13 (52%, CI 31–73) of 25 patients meeting the ML‐SADDLE‐20.

#### 4.7.2 Ustekinumab

Ustekinumab is a monoclonal antibody that targets the p40 subunit of IL‐12 and IL‐23, which both mediate SLE pathogenesis. In a multicenter, double‐blinded phase II trial for SLE, the primary outcome of SRI‐4 at week 24 was met, with 62% of patients in the ustekinumab group compared to 33% of those in the placebo group.^74^ The percentage difference between treatment arms was 28% (CI 10–47, *P* = 0.006). Post hoc analysis of the CLASI‐50 response at week 24 demonstrated superiority of ustekinumab over placebo (64.1% vs 29.9%, *P* = 0.032) with a difference of 18% (CI −1 to 37). The most common adverse events were upper respiratory and urinary tract infections, nasopharyngitis, and headache. The phase III study was terminated because the primary endpoint at week 52 of the SRI‐4 response rate was not met nor were the secondary endpoints, which included skin response reported as CLASI‐50.^75^

#### 4.7.3 Fenebrutinib and branebrutinib

Fenebrutinib is a selective Bruton's tyrosine kinase (BTK) inhibitor that targets B cells and myeloid cells implicated in the pathogenesis of SLE.^76^ In a phase II trial (NCT02908100), fenebrutinib did not meet its primary endpoint of SRI‐4 response rates at week 48, with 51% for fenebrutinib 150 mg qD (*P* = 0.37 vs placebo), 52% for fenebrutinib 200 mg BID (*P* = 0.34 vs placebo), and 44% for placebo. Exploratory subgroup analysis of the SRI‐4, which included CLASI responsiveness, revealed no significant difference between the fenebrutinib‐treated group and placebo.

Branebrutinib is another selective BTK inhibitor that recently completed a phase II trial (NCT04186871). The primary endpoint was the percentage of patients who achieved at least a 50% decrease in the modified CLASI activity score from baseline (mCLASI‐50) and corticosteroid dose <10 mg/day at weeks 20 and 24. Preliminary analysis of the primary endpoint showed non‐superiority of branebrutinib compared to placebo with −27.8 response difference (CI −77.5 to 21.9).

#### 4.7.4 RSLV‐132

The fusion protein RSLV‐132 consists of a catalytically active RNase fused to the Fc domain of IgG1.^77^ This novel medication degrades circulating RNA, thereby inhibiting immune activation through the TLR and IFN pathways. A phase II trial of RSLV‐132 in SLE patients with active cutaneous lesions did not demonstrate superiority in mean CLASI score change from baseline in the treatment arm ( day 85: mean ‐6.2 (SD=6.7); day 169: ‐6.2 (8.5)) compared to placebo (day 85 mean: ‐6.5 (6.2); day 169: ‐5.7 (7.0)). Composite endpoint analysis showed that treatment arm subjects with a high SLEDAI had a higher rate of SRI‐4 responses (62% vs 44%) and a higher rate of BICLA responses (23% vs 11%) than placebo group particpants.^78^

#### 4.7.5 Other cytokines

Several therapies explored in SLE ceased further development after unfavorable phase II study results: BT063, a monoclonal antibody targeting IL‐10 (NCT02554019); vobarilizumab, a nanobody against IL‐6 (NCT02437890); PF‐04236921, an IL‐6 monoclonal antibody (NCT01405196)^79^; and avizakimab, an anti‐IL‐21 monoclonal antibody (NCT03371251).

## 5 CONCLUSION

The current treatment options for CLE are notably inadequate, often necessitating treatment escalation without sufficient control of disease activity. This gap in management significantly impacts quality of life for CLE patients, underscoring a critical need for more optimal treatments. Our existing therapeutic armamentarium consists primarily of broad‐spectrum immunosuppressive agents that carry a significant risk of systemic side effects. A crucial ongoing objective is establishment of clinical trials dedicated to CLE for properly tailored inclusion criteria and endpoints focused on cutaneous parameters. Differentiation of CLE from SLE will enable precise skin assessment and improve medication accessibility for patients with exclusively dermatologic disease. Despite historical challenges, recent phase II and III trials for CLE have shown encouraging results for treatments targeting the IFN, pDC, and TLR pathways.

Anifrolumab, an anti‐IFNAR1 monoclonal antibody, has shown optimistic outcomes in phase II and III SLE trials, although its efficacy in CLE necessitates further research. Initial improvements occurred as early as week 8 and were sustained through week 52. Smaller‐scale studies have implicated its utility in treatment‐resistant CLE patients, although a trial specific to CLE patients is essential for comprehensive evaluation. The ongoing phase III trial (NCT04877691) is of particular interest in the exploration of subcutaneous administration instead of monthly intravenous infusions.

Litifilimab, which blocks BDCA2 receptors on pDCs, has marked a significant milestone in CLE research in the LILAC Part B study. This study sets a precedent for future CLE research by evaluating therapeutic results separately in CLE and SLE, and by modeling skin‐specific design. This trial included two major CLE subtypes, SCLE and CCLE, utilized rigorous photovalidation for characterizing subtypes and severity, and employed the CLASI as a validated outcome measure for its primary endpoint. The ongoing phase III CLE trial (NCT05531565) is being closely monitored as a potential candidate for the first biologic approval for CLE.

Given the efficacy of litifilimab, daxdilimab, which also targets pDCs but through a different mechanism of action by binding to the ILT7 receptor, is anticipated to yield comparable positive outcomes. Daxdilimab has shown promise in early trials with a favorable safety profile and is currently in a phase II CLE trial.

Another investigational agent is deucravacitinib, a TYK2 inhibitor involved in IFN‐1, IL‐10, IL‐12, and IL‐23 signaling. After a phase II SLE trial promising results with CLASI measured as a secondary endpoint, investigation of deucravacitinib continues in a dedicated phase II CLE trial. This agent is already approved for psoriasis and is especially appealing due to its positive safety profile, lack of the black box warning present in other JAK inhibitors, and oral route of administration, which may be preferable for some patients over injectable treatments.

Iberdomide exhibits a multifaceted approach to target CLE pathogenesis through suppression of pDCs, IFN‐1, B cells, and anti‐dsDNA antibodies while enhancing IL‐2 and regulatory T cells through degradation of Ikaros and Aiolos. In a phase II SLE trial, SCLE patients treated with iberdomide showed improvement and CCLE patients trended toward improvement. These findings suggest that different CLE subtypes may have varied responses to treatment. Iberdomide has unfortunately not been tested in a skin‐specific trial, and no phase III trials are currently planned.

Ultimately, this review underscores the challenges in developing effective therapies for CLE. Establishment of CLE‐focused clinical trials, use of appropriate skin inclusion criteria, and employment of validated skin measures is crucial to achieve this goal. The CLASI is a valuable tool that captures impactful changes in disease severity, although the evaluation of the CLA‐IGA as per FDA guidance is underway. Due to its heterogenous presentation, CLE patients require a tailored approach to treatment, with future research focusing on selective biomarkers and pathways to personalize therapy. This individualized approach could be pivotal in identifying potential responders and optimizing treatment efficacy for CLE patients.

## FUNDING INFORMATION

This manuscript was funded by the National Institutes of Health‐USA (NIH‐USA) grants R01AR071653 (to V.P.W.) and the United States Department of Veterans Affairs Merit Review BX005921‐01 (Veterans Health Administration, Office of Research and Development and Biomedical Laboratory Research and Development, to V.P.W.).

## CONFLICT OF INTEREST STATEMENT

V.P.W. has grants from Celgene, Janssen, Pfizer, Biogen, Gilead, Corbus Pharmaceuticals, Genentech, AstraZeneca, Viela, Syntimmune, Amgen, Regeneron, Argenx, CSL Behring, Ventus, q32 Bio, BMS, and Horizon and has consulted for Celgene, Genentech, Janssen, Lilly, Pfizer, Biogen, BMS, Gilead, Amgen, Medscape, Nektar, Incyte, EMD Sorona, CSL Behring, Principia, Crisalis, Viela Bio, Argenx, Kwoya Kirin, Regeneron, Principia, AstraZeneca, Abbvie, Octapharma, GSK, Astra‐Zeneca, Cugene, UCB, Corcept, Beacon Bioscience, Rome Pharmaceuticals, Horizon, Gilead, Merck, Kezar, Sanofi, Bayer, Akari, Calyx, and Cabaletta Bio. The University of Pennsylvania owns the copyright for the CLASI. The authors have no other relevant affiliations or financial involvement with any organization or entity with a financial interest in or financial conflict with the subject matter or materials discussed in the manuscript apart from those disclosed.
